# Carbon Monoxide Intoxication Leading to Crush Syndrome and Acute Renal Failure: A Case Report

**DOI:** 10.7759/cureus.72693

**Published:** 2024-10-30

**Authors:** Sümeyra Koyuncu, Mehmet Yasin Turkmen, Nazmiye Serap Bicer, Hilal Sipahioglu

**Affiliations:** 1 Nephrology, Kayseri State Hospital, Kayseri, TUR; 2 Internal Medicine, Kayseri City Hospital, Kayseri, TUR; 3 Intensive Care Unit, Kayseri City Training and Research Hospital, Kayseri, TUR

**Keywords:** acute renal failure, carbon monoxide poisoning, crush syndrome, hyperbaric oxygen therapy, rhabdomyolysis

## Abstract

Carbon monoxide (CO) poisoning is a common occurrence, typically resulting from exposure to CO emissions in enclosed spaces. CO is a colorless, odorless, and tasteless gas.

In this case report, we present a 52-year-old male patient who developed crush syndrome and acute kidney injury following carbon monoxide (CO) intoxication. The patient was found at home with loss of consciousness and seizures and was brought to the emergency department. A diagnosis of CO poisoning was made, and the patient was treated with hyperbaric oxygen therapy and supportive care. The clinical course, treatment outcomes, and comparison with the existing literature are detailed.

## Introduction

Typically resulting from exposure to carbon monoxide (CO) emissions in enclosed places, CO poisoning is a common occurrence. CO is a colorless, odorless, and tasteless gas that binds to hemoglobin, reducing its oxygen-carrying capacity and leading to tissue hypoxia [1]. Symptoms of CO poisoning can range from headaches, nausea, and dizziness to coma. СΟ poisoning is estimated to occur in 50,000 people annually in the United States [2].

Crush syndrome is characterized by damage to muscle tissue exposed to prolonged pressure and its systemic effects. It is commonly associated with trauma or entrapment injuries and is linked to myoglobinuria, acute kidney injury, and coagulation disorders. Intrarenal kidney injury in the setting of crush-related injury is typically due to rhabdomyolysis. The characteristic manifestation of rhabdomyolysis-related acute tubular necrosis (AТΝ) is dark red, brown, or black urine [3,4]. The release of myoglobin and other toxic substances from damaged muscle cells can accumulate in the kidneys [5].

CO binds to hemoglobin more readily than oxygen, forming carboxyhemoglobin and preventing oxygen binding, which can result in muscle hypoxia and rhabdomyolysis [6]. Reports of CO poisoning leading to crush syndrome are rare, and further investigation is needed to understand the pathophysiological relationship and the effects of CO on muscle tissue and systemic health. This report discusses the clinical presentation, treatment, and outcomes of a patient with CO poisoning who developed crush syndrome.

## Case presentation

A 52-year-old male was found at home with impaired consciousness and seizures and was transported to the emergency department. On admission, the patient’s Glasgow Coma Scale score was 10, with vital signs showing blood pressure of 123/89 mmHg, pulse rate of 89 beats per minute, temperature of 37.7°C, and saturation levels indicating hypoxemia (level of SpO_2_:85). Arterial blood gas parameters were as follows: potential of hydrogen (pH)=7.36, partial pressure of carbon dioxide (pCO_2_)=38.1 mmHg, bicarbonate (HCO_3_)=21.7 mEq/L, lactate=2.9 mmol/L, partial pressure of oxygen (pO_2_)=60 mmHg, oxygen saturation (SpO_2_)=85 (%), carboxyhemoglobin (COHb)=16 (%). According to the patient's history, the patient’s home had a malfunctioning stove emitting smoke upon his return.

On examination, the patient exhibited normal cardiac and respiratory findings. The abdominal exam was unremarkable, with no signs of rebound tenderness or guarding. On physical examination of the extremities, there was mild swelling in the lower limbs, but pulses were palpable, and the patient was diagnosed with CO poisoning. Initial management included hyperbaric oxygen therapy (2.5 atmospheres absolute), followed by 4-6 L/min of nasal oxygen.

Table 1 presents the laboratory tests.

**Table 1:** Laboratory parameters

Laboratory markers	Values		Reference values	Units (SI)
	1st day	5th day		
Blood urea nitrogen	38 ­	15	6-20	mg/dl
Creatinine	2.7 ­	1.2	0.5-1.2	mg /dl
Sodium	151­	135	136-145	mmol /l
Potassium	5.7­	3.5	3.5-5.1	mmol/l
Total bilirubin	1.1	0.9	0-1.4	mg/dl
Direct bilirubin	1.0	0.3	0-0.3	mg/dl
Total protein	5.1	5	6.4-8.3	gr/dL
Albumin	4.2	3.8	3.5-5.2	gr/L
Alkaline phosphatase	99	95	44-130	u / L
Lactate dehidrogenase	345 ­	240	135-250	u / L
Aspartate aminotransferase	431 ­	50	0-40	u / L
Alanine aminotransferase	195 ­	40	0-41	u / L
Gamma-glutamyl transferase	76­	55	10-71	u / L
C-reactive protein	174­	20	0-5	mg /L
Hemoglobin	16	15.1	12-16	g /dl
Platelet	319000	250000	130-400	µ /L
White blood cell	12.99x10^9^	8x10^9^	4.8-10.7x10^9^	/liter
Creatine kinase	43120 ­	570	0-500	ug/L

Due to oliguria, the patient was admitted to the nephrology service for rhabdomyolysis and acute renal failure, and intravenous hydration was initiated. After two days, despite an increase in urine output, the patient developed swelling, heat loss, and pain in the left leg, raising concerns for compartment syndrome. A Doppler ultrasound was performed, which was normal. The patient was treated with IV mannitol and IV bicarbonate. The patient received one liter of isotonic saline alternating with 1 liter of half-normal isotonic saline plus 50 mEq of sodium bicarbonate and 50 mL of 20 percent mannitol.

The patient's clinical and laboratory values improved, leading to his eventual discharge.

## Discussion

The decrease in arterial oxygen transport and the left shift of the oxyhemoglobin dissociation curve explain the acute hypoxic symptoms seen in CO poisoning. Children, the elderly, and active individuals are particularly susceptible, and exercise, stress, and anemia can exacerbate this risk. The brain and heart, due to their high oxygen demand, are especially vulnerable to CO-induced hypoxia [7]. Severe CO poisoning can result in neurological symptoms such as stroke or coma, as well as metabolic complications like myocardial ischemia, ventricular arrhythmias, pulmonary edema, and severe lactic acidosis [8].

Compartment syndrome results from increased tissue pressure in a confined space, compromising circulation and function. This condition occurs when pressure exceeds a level sufficient to reduce capillary perfusion. Inadequate tissue perfusion leads to insufficient oxygenation of the affected muscles and nerves. Characteristic symptoms include disproportionate pain, particularly on passive stretching of the fingers. Pain typically does not improve with rest or analgesics [9].

CO poisoning is among the most prevalent types of poisoning globally. While direct muscle toxicity from CO exposure is rare, compartment syndrome associated with CO intoxication has been reported. This is probably related to hypoxia-induced necrosis of muscle tissue [6]. This case highlights the importance of rapid and effective management of crush syndrome and acute renal failure.

Hyperbaric oxygen therapy is recommended for severe CO poisoning as it helps displace CO from hemoglobin and reduces tissue hypoxia [10]. A multidisciplinary approach is essential for managing complications such as rhabdomyolysis and acute renal failure. Intravenous fluids, mannitol, and bicarbonate can support renal recovery and mitigate the systemic effects of muscle damage [11]. The treatment strategies applied in this case played a crucial role in the patient’s recovery.

## Conclusions

This case demonstrates the challenges of managing severe complications arising from CO poisoning. It underscores the importance of a multidisciplinary approach and effective treatment strategies in improving patient outcomes. Prompt identification and management of similar cases can optimize the treatment process and enhance patient recovery.
